# Assessing female call responses to syllable level details in song

**DOI:** 10.3389/fpsyg.2025.1523105

**Published:** 2025-03-12

**Authors:** Nora H. Prior, Adam R. Fishbein, Esther Martinez Garcia, Savannah Clough, Mary R. Elson, Gregory F. Ball, Robert J. Dooling

**Affiliations:** ^1^Department of Psychology, Cornell University, Ithaca, NY, United States; ^2^Department of Psychology, University of Maryland, College Park, MD, United States; ^3^Neuroscience and Cognitive Science Program, University of Maryland, College Park, MD, United States; ^4^Department of Animal Sciences, University of Maryland, College Park, MD, United States

**Keywords:** vocal communication, song playback, acoustic fine structure, mate assessment, preference

## Abstract

Mate choice is a critical decision-making process, having lasting impacts on an individual’s time, energy, and reproductive success. Across songbirds, females are generally assumed to prefer higher song rates, greater complexity, and higher quality performances; however, there is growing evidence implicating syllable level details in songbird communication. Here, we build on our previous psychoacoustic results to ask whether female zebra finches use the kinds of syllable level details that they are capable of hearing. Female zebra finches produce calls during male songs as a component of courtship. These calls can be leveraged to explore how females assess and interact with male songs. To test whether syllable level details are behaviorally relevant in a courtship context, we quantified female call responses to manipulated songs in four experiments. First, we validated that our playback procedure elicited robust calling responses from females (Exp 1). Next, we found that females decreased calling to songs where syllables were spectro-temporally reversed (REVERSAL), but did not respond differently if the syllable order was manipulated (SHUFFLED). Females also modulated their calling when experimental songs were composed of natural rendition-to-rendition variation in song syllables (RENDITION) relative to songs consisting of a single repeated rendition (FIXED) (Exp 2). Furthermore, we found that females decreased calling responses even when only a portion of syllables were spectro-temporally reversed (Exp 4). Across these experiments, we also report the striking extent to which females habituated to a male’s song (Exps 3 and 4). To maximize female responses, we tried adjusting the paradigm in Exps 3 and 4 to increase female calling. However, our adjustments had minimal effects, consistent with the notion that females rapidly decreased calling in response to a given males’ stimuli. Altogether, our results contribute to growing evidence that syllable level details in birdsong are behaviorally relevant, and, perhaps more importantly, demonstrate that birds’ enhanced ability to discriminate acoustic fine structure as shown in psychophysical tests plays a role in communication.

## Graphical abstract

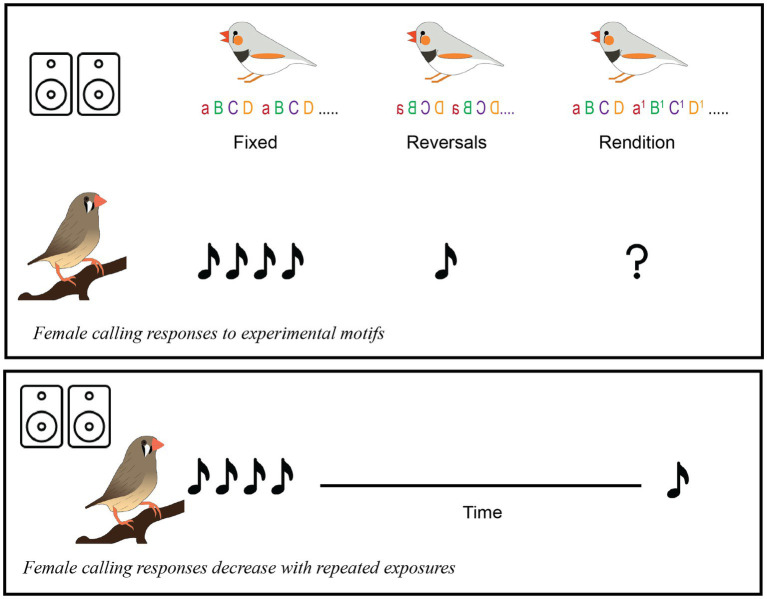

## Introduction

Mating and reproduction are energetically costly and time-intensive, have long-lasting impacts on individuals, and are processes heavily shaped by natural and sexual selection (Bateson, 1983; Rosenthal, 2017). Understanding the behavioral and neurobiological mechanisms behind these mate-choice decisions has long fascinated researchers across biological disciplines, from evolution to neuroscience. However, even for well-studied species, such as the zebra finch (*Taenopygia gutatta*), research suggests that mate preferences can be weak, variable, and have low repeatability (Forstmeier and Birkhead, 2004; Riebel, 2000; Rosenthal, 2017).

In songbirds, females are generally assumed to prefer higher song rates, greater complexity, and higher quality performances (Hauber et al., 2010; Rose et al., 2022). However, the process of mate assessment varies greatly across songbird species. For some species, courtship songs may be used to attract females to a territory with males and females interacting very little while females assess a male’s song (Byers and Kroodsma, 2009; Catchpole and Slater, 2008; Searcy, 1984). For other species, courtship involves robust interactive vocal exchanges (Coleman and Fortune, 2018; D’Amelio et al., 2017). Even in species where the females do not sing, many females produce calls during male songs as an important component of courtship (Amy et al., 2015; Chen et al., 2017; Prior, 2020).

In this study, we investigated female assessment of male song in zebra finches by quantifying female calling responses to passive playback of male song. Zebra finches are a well-studied avian system, but female preferences are often weak, variable, and/or dynamic (Forstmeier and Birkhead, 2004; Rosenthal, 2017). Across lab and field studies, there is evidence that zebra finches tend to prefer more complex and longer songs (Clayton and Pröve, 1989; Collins, 1999; Holveck and Riebel, 2007; Woodgate et al., 2012). Females also often prefer the high-quality performance of directed song over undirected song (for both their mate and unfamiliar individuals) (Chen et al., 2017; Wall and Woolley, 2024; Woolley and Doupe, 2008). While female preferences for directed song could be explained by attention to global features (e.g., stereotypy), these preferences could also be explained by the quality of individual syllables (Woolley and Doupe, 2008). Importantly, the most robust preferences females appear to have are for their mates song, potentially independent of what syllables are present (Woolley and Doupe, 2008; Woolley and Woolley, 2020).

While focusing on global song features, such as complexity, aligns with frameworks of signal selection across species (Rosenthal, 2017), global features also stand out to us as human listeners. Because we easily distinguish zebra finch songs and individual males by rhythm, syllable types, and motif patterns, it is easy to assume female zebra finches do the same. However, there is growing evidence suggesting that females may be more attuned to details within individual syllables (Geberzahn and Derégnaucourt, 2020; Geberzahn et al., 2021; Lawson et al., 2018; Prior et al., 2018). While our past work has shown that syllable level details are considerably more discriminable than global features (Lawson et al., 2018; Stennette et al., 2023), it remains unclear whether easily discriminable features reflect what is behaviorally relevant.

We have previously shown that zebra finches are able to discriminate fine acoustic details of their vocalizations an order of magnitude better than humans can (Dooling and Prior, 2017; Prior et al., 2018). We have shown that zebra finches, when listening to song, find syllable level details more salient than the order of syllables (Lawson et al., 2018; Stennette et al., 2023), and that they can easily discriminate the smallest produced acoustic differences in a syllable or motif from moment-to-moment (renditions) (Fishbein et al., 2021). Evidence from other labs also emphasize the importance of syllable level details. For example, zebra finches use syllable level information (not which syllables are present) to identify individuals (Geberzahn and Derégnaucourt, 2020; Geberzahn et al., 2021). Similarly zebra finches use fine acoustic signatures specific to each call category to recognize individuals (Elie and Theunissen, 2018). Finally, zebra finches are highly sensitive to fine acoustic details and may be able to extract many types of behaviorally relevant information previously overlooked (Dooling and Prior, 2017; Prior et al., 2019; Prior et al., 2018).

Across the four experiments in this study, we asked whether females modulate their calling based on syllable level details within a song rather than global features. We used experimentally manipulated songs to target differences in syllables in motifs. Across the experiments, we focused on four types of manipulations (motif conditions): FIXED—where the same exact motif is repeated multiple times to produce a song bout, SHUFFLED—where the same exact syllables are repeated but in different orders across motifs within a song bout, REVERSAL—where individual syllables are spectro-temporally reversed, and RENDITION where syllables from different naturally occurring motifs are used to generate each motif in a song bout.

Based on previous research, we would expect that females are insensitive to the order of syllables within a motif, and therefore we predicted that females would call similarly to FIXED and SHUFFLED motifs (Lawson et al., 2018; Stennette et al., 2023). Because zebra finches are easily able to discriminate syllable level acoustic differences across renditions (Fishbein et al., 2021), we predicted that females may have differing responses to FIXED and RENDITION conditions. Finally, we predicted that females would call less to the REVERSAL condition. Not only are syllable reversals highly discriminable, but reversing syllables modify the acoustic features that support individual and species identity. Finally, by reversing some, but not all the syllables in a song (Exp 4), we tested how sensitive zebra finches are to only a few syllable distortions in song. Altogether, our results across these four experiments contribute to growing evidence that acoustic fine structure in individual song syllables is behaviorally relevant, supporting the notion that the remarkable ability to discriminate acoustic fine structure plays a role in communication.

Through the course of running these experiments, we also worked to optimize female responses and discovered that females rapidly habituate to song playbacks from a given male. Across the four experiments, we tried various adjustments to mitigate this. Overall, our adjustments had minimal effects. In the discussion, we also highlight some of the challenges and potential strategies for using call responses in this paradigm.

## General methods

### Experimental approach

**Figure 1:**
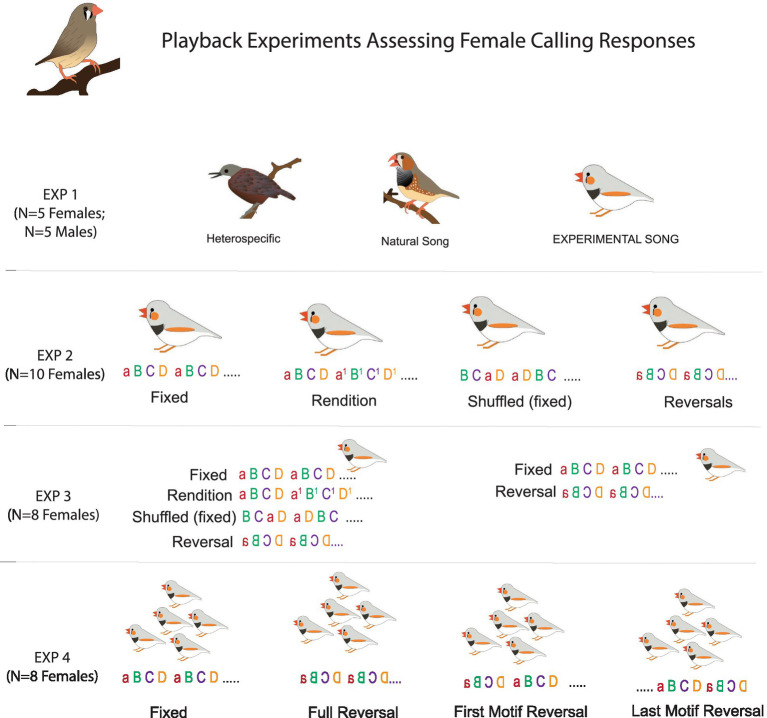
Schematic of experimental playback conditions. Song syllables are indicated with letters (a, b, c, d) and two motifs for each song bout are depicted.

**Figure 2:**
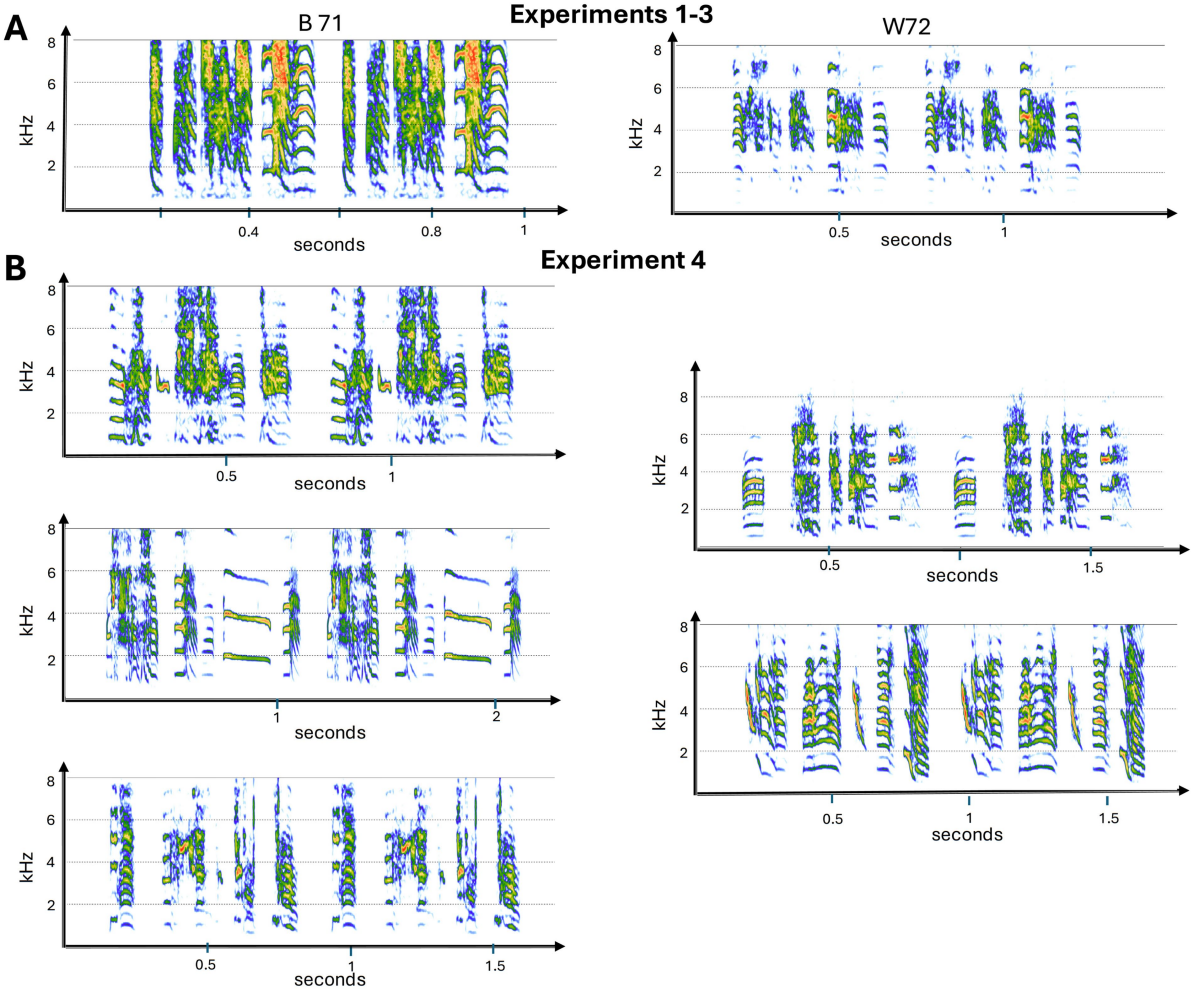
Spectrograms of the male songs used in the experiments. Panel **(A)** shows songs used in Experiment 1–3. W72 was used in Experiment 1–2, and then B71 was used in Experiments 2–3. All 5 songs in panel **(B)** were used in Experiment 4. Two motifs of each song bout are depicted. See Figure 1 and Table 1 for further details about the different approaches used across the experiments. Spectrograms were made using the R package Seewave version 2.2.3 (Sueur et al., 2008).

Female calling during male songs is a prominent component of courtship. While the function of these female calls remains largely unstudied, these calling responses have been used as an indicator of female song and mate preferences (Chen et al., 2017; Clayton, 1988; Dunning et al., 2014; Nagle et al., 2002). We used female calling in response to experimental song bouts to assess the functional significance of syllable level acoustic variation across four experiments (Table 1 and Figures 1, 2). An overview of these experiments is presented below.

**Table 1:** Differences in playback procedure across the four experiments.

Experiment	Sample size	Duration (min)	Motif conditions	# Sessions	# Motifs/song	# Male song/exp	Song duration (s)	# Songs/playback	Playback duration	Inter-song silence(s)	Notes
*Exp 1*	5 F & 5 M	5	Natural ZF song Experiment ZF song Antbird	3	4	1	2.2	6	5	40	The order of motif conditions was counterbalanced by session across individuals.
*Exp 2*	10F	5	Fixed Reversal Shuffled Rendition	4	4	2	2.9	6	5	40	The order of motif conditions was counterbalanced by session across individuals.
*Exp 3a*	8 F	8.5	Fixed Reversal Shuffled Rendition	1	8	1	6.4	8	11.3	45	All motif conditions were presented in a single playback. However, there were 4 versions of this playback, and the order of motif conditions within a playback was counterbalanced across individuals.
*Exp 3b*	8F	8.5	Fixed Reversal	2	8	1	Same as exp 3a	8	11.3	45	Both motif conditions were presented in a single playback; 2 versions of this were made so the order of motif conditions was counterbalanced across individuals. Females were tested twice. Once they also had access to a visual stimulus (an image of a zebra finch behind a cheese cloth). This was counterbalanced across sessions.
*Exp 4*	8F	8.5	Fixed Reversal Reversal (first motif) Reversal (last motif)	4	8	5	5.2 5.9 6.1 6.2 7.7	10	8.5	45	The order of motif conditions was counterbalanced by session across individuals.

This table highlights the differences across experimental design. We modified the behavioral paradigm across the four experiments as we worked to increase female responses.

#### Experiment 1: female call responses to experimental song bouts

First, we validated that a brief song playback procedure elicited calling responses from females. To do this, we exposed males (*N* = 5) and females (*N* = 5) to three different playback conditions:

1. Natural zebra finch song: Unmanipulated recordings of zebra finch song, including introductory notes, natural timing (inter-syllable and inter-motif silences), and natural background noise.
2. Experimental zebra finch song: A single motif repeated four times to generate a song bout. Because our goal was to focus on the role of song syllables specifically, we removed global features of songs that could be behaviorally relevant. We removed all the introductory notes, and to remove motif-motif variation in the timing of syllables, we replaced all the inter-syllable intervals with consistent silences based on a single rendition for each male.
3. Chestnut-backed antbird (*Myrmeciza exsul*) vocalizations: We used antbird vocalizations to assess zebra finch calling in response to a non-social context. Zebra finches and antbirds are allopatric, and antbird vocalizations are acoustically different than zebra finch vocalizations (antbird: Strong, 2017).

Each bird heard all three conditions, with at least 24 h between tests. The order of presentation was counterbalanced across birds.

If zebra finch call responses to male song are predominately related to courtship interactions, we would predict females would call to male zebra finch song, but that males may not. We also needed to validate that experimental song bouts, focusing on song syllables alone, would elicit robust responses from females even though they lacked introductory notes and other global features typical of male song.

#### Experiment 2: female call responses to syllable level manipulations within song

After validating our general approach, we aimed to test whether females (*N* = 10) respond differently to songs based solely on manipulations to song syllables. We quantified female call responses to four ‘motif conditions’, reflecting different types of manipulations to syllables.

1. FIXED: a single motif was repeated to generate a song bout.
2. RENDITION: four separate renditions of a motif were used to generate a song bout.
3. REVERSAL: a single motif, with each syllable spectro-temporally reversed but kept in its natural position, was repeated to generate a song bout.
4. SHUFFLED: a single motif was used to generate a song bout and the order of the syllables within the motif was randomly rearranged in each motif.

Each bird heard all four conditions, with at least 24 h between tests. The order of presentation was counterbalanced across birds. All females were run on these motif conditions for two different males’ songs (in total each female was run 8 times).

We predicted that females would call: (1) similarly between SHUFFLED and FIXED motif conditions; (2) differently to RENDITION vs. FIXED conditions, and (3) less to the REVERSAL condition.

#### Experiment 3: assessing female call responses to multiple motif conditions within a single playback

Based on Exp 2, we used song B71 (Figure 2) which produced robust female responses. We used this song with the same motif conditions as Exp 2, but with adjustments to the playback approach with the goal of minimizing confounds and increasing female responses. A different set of females was used in this experiment.

- **Experiment 3a**. First, we used the same set of motif conditions (FIXED, RENDITION, REVERSAL, SHUFFLED) as Exp 2, but presented in a single playback (Table 1 and Figure 1). We also increased the length of each song bout from 4 motifs to 8 motifs and increased the overall duration of the playback, with the hope that this would increase female calling responses. To counterbalance the order of motif conditions across individuals, we created four versions of this playback where the order of motif conditions differed.
- **Experiment 3b**. As a cleaner test of our adjustment to the paradigm (including multiple motif conditions in a single playback), we next focused on only two motif conditions (FIXED and REVERSAL), to see if we could replicate our results from Exp 2. Like above, both motif conditions were presented in a single playback and two versions of this were made so the order of motif conditions was counterbalanced across individuals. In addition, females were tested twice. Once they also had access to a visual stimulus (an image of a zebra finch behind a cheese cloth). The presence of a visual stimulus was counterbalanced across the two sessions. Based on previous research, we predicted a visual stimulus could increase call responses (Perez et al., 2015). The sessions were conducted on the same day several hours apart.

#### Experiment 4: assessing the degree of female sensitivity to reversals

To determine whether females are sensitive to only a portion of syllables within a song, we leveraged the fact that reversals cause a decrease in female responses. Here, we used four motif conditions:

1. FIXED: As described above—all eight motifs in the song bout were the same.
2. REVERSAL: As described above—all syllables were spectro-temporally reversed in place. All eight motifs were the same.
3. FIRST MOTIF REVERSAL: The syllables in the first motif were spectro-temporally reversed, but the rest of the syllables were the same as the FIXED condition. Thus only 1/8th of the syllables were manipulated.
4. LAST MOTIF REVERSAL: The syllables in the last motif were spectro-temporally reversed, but the rest of the syllables were the same as the FIXED condition. Thus only 1/8th of the syllables were manipulated.

Based on our results from Exp 2–3, we used a hybrid approach in this experiment. We used songs from five new males. Each playback contained only one motif condition but used songs from all five males. For each playback, females only heard two songs from each male. The motif conditions were then presented across four sessions with 2–3 days rest in between each session.

### Subjects

Zebra finches are opportunistic breeders and form life-long pair bonds (Prior and Soma, 2015; Zann, 1996). Thus, we took precautions to ensure females were breeding ready and motivated to engage in courtship behaviors. Birds were housed under long days (12 L:12D), kept in same-sex flocks, and given high-quality environmental conditions (e.g., seed and water *ad lib*).

In total, we ran 23 birds across the four playback experiments including five male zebra finches and 18 female zebra finches. For Exp 1, we used five males and five females. Then in Exp 2, we tested 10 females. Four females were tested in both Exp 1 and 2. Finally, we ran eight females in Exp 3 and Exp 4 (one of whom had also been used in Exp 2). In total, we used songs from seven different males. All birds were young adults (between 6 months and 2 years) (also see Figure 2).

### Playback procedure

For each playback test, the experimental song bouts were played from a Bluetooth (iLuv AUD mini) speaker connected to an iPad Mini placed about 30 cm from the cage. Stimuli were played back at ~75 db, approximately 30 cm from the cage (where the bird was positioned). See Table 1 for details on the duration of the song bouts and number of songs/motifs included in the playback for each experiment.

The procedure was as follows: An individual bird was brought from the colony room and allowed to freely enter the testing cage. The researcher then started the audio recording, left the room, and shut the door. Playback was then started using the iPad Mini. See Table 1 for details on the playback stimuli and duration of each playback. At the end of the playback period, the playback program was stopped. Then the researcher would enter the room, stop the recording, and returned the female to her home cage in the colony. When females were exposed to multiple playbacks in an experiment, the order of playback stimuli was counterbalanced across birds.

### Preparation of stimuli

We recorded directed song from seven zebra finches in a foam-covered room. Recordings were made using a tie-clip microphone (AKG C417) and a Zoom F8 multitrack field recorder (sampling rate of 44.1 kHz). These were males that females had never interacted with directly, but they had been housed in the same colony room.

Our method of sample preparation has been described previously (Fishbein et al., 2021). Songs were viewed in Adobe Audition (ver: 2015.2), and motifs were selected that did not have competing background noise (i.e., wing fluffs, cage noises, and female calls, etc.) (Figure 2). Using Adobe Audition, motifs were high-pass filtered with a cutoff frequency of 350 Hz. After individual syllables were extracted, motif stimuli were generated in MATLAB (MathWorks, Natick, MA). Experimental song bouts were built from extracted syllables, prepared with 5 ms cosine rise/fall times. Consistent rise/fall times are necessary to preserve the acoustic features of syllables following inter-syllable intervals of complete silence. In addition, motifs were constructed with consistent inter-syllable intervals based on the natural motif and a consistent interval of silence was used between motifs within the song bout. These adjustments normalize other global features of the song which females may attend to when assessing songs. For the REVERSAL condition, syllables were spectro-temporally reversed in MATLAB (fliplr). For the RENDITION condition, syllables were extracted from consecutive motifs to build the song bout (Fishbein et al., 2021), and for the SHUFFLED condition, the positions of syllables were assigned randomly.

### Scoring call behavior

All the recordings were analyzed using Adobe Audition. The number of calls was counted prior to the start of the recording, during the period of playback (both within song bouts and outside of song bouts), and for post-playback. In Exp 1 and 2, we scored calls for ~2.5 min pre and post playback, and for Exp 3 and 4, we scored calls for ~1 min pre and post playback. Because the microphone was closer to the female, the female vocalizations were much louder than the song playback, and we were able to use these level differences to identify female calls within male song. Overall, we found similar results regardless of whether we used call rate, total calls during playback, or calls during song as our dependent variables. Additionally, we saw similar results regardless of whether we corrected for calls produced prior to playback.

### Analysis

Statistical analyses were carried out in R (v.4.3.1, R Foundation for Statistical computing), using linear-mixed models [function lmer from the lme4 package (Bates et al., 2015)] for call rate, or generalized linear-mixed models (glmer with Poisson distribution) for call count data. Calling responses (either number of calls or call rate) was used as the response variable and bird ID was included as a random factor. The main explanatory variable of interest across experiments was Motif or Playback Condition (e.g., fixed, reversal, shuffled). In Exp 2, Song was also used as an explanatory variable (e.g., B71, W72). In Exp 3, Visual Condition (with or without a visual stimulus) was tested as well. Additionally, in Exp 3 and Exp 4, we asked whether there was an order effect independent of other explanatory variables that shaped call activity. Therefore, we included Session of testing as an explanatory variable (e.g., first, second, third, fourth). Prior to interpretation, we checked the validity of each model by plotting the distribution of the residuals. Follow up tests were done on lmer or glmer models using pairwise comparisons with estimated marginal means (emmeans) [package emmeans (Lenth, 2024)].

## Results

### Females call to experimental songs (Experiment 1)

**Figure 3:**
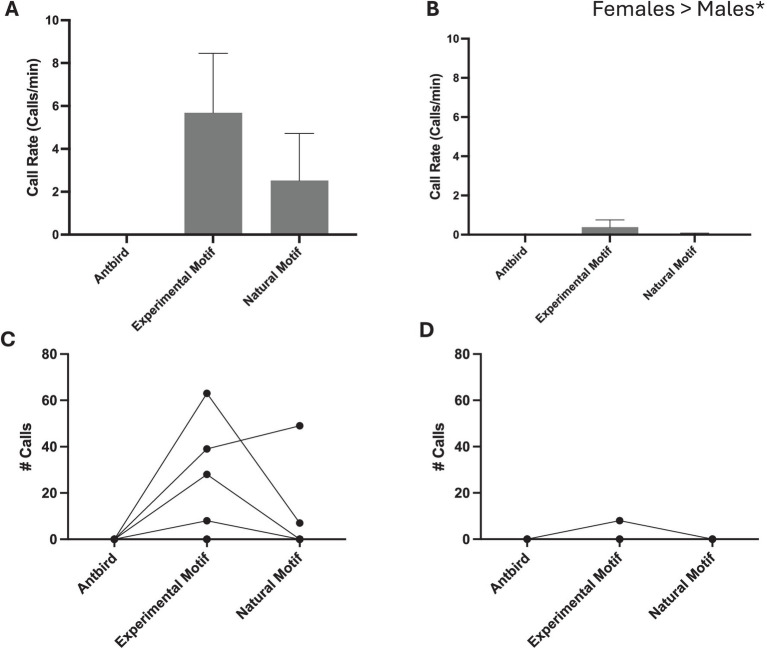
Calling responses of females **(A,C)** and males **(B,D)**. Individual data is presented for females **(C)** and males **(D)**. Overall females called more than males did to zebra finch song stimuli, and females called similarly to Experimental and Natural song.

Overall, females called more to song than males did across all stimuli (Mean ± SD. *Female* 8 ± 12 calls and *Male* 1 ± 2.6 calls; one-tailed t-test *p* = 0.0493) (Figure 3). No birds called in response to heterospecific playback. Three out of five females called in response to playback (three in response to the experimental song playback and two in response to the natural song playback). One male called in response to the experimental playback and one in response to the natural playback. Importantly, females called similarly to both the normal song bout and the experimental song bout (Mean ± SD. *Experimental Motif* 26 ± 26.6 calls and *Normal Song* 11.2 ± 21.3 calls; one-tailed, paired t-test *p* = 0.144).

### Female calling is modulated by variation in song syllables (Experiment 2)

**Figure 4:**
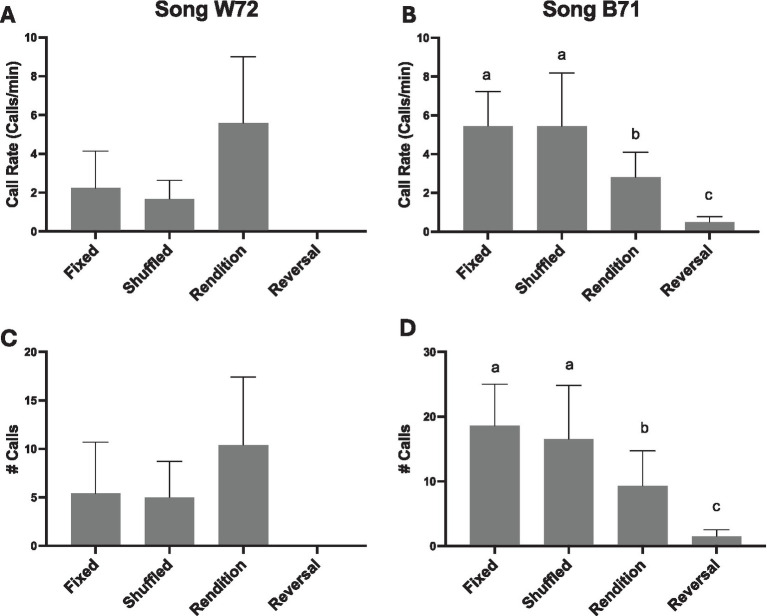
Female call rate in response to the four motif conditions (FIXED, RENDITION, REVERSAL, and SHUFFLED) for songs from two different males: Song W72 **(A,C)**, and Song B71 **(B,D)**. The top panels **(A,B)** show the results for call rate based on total calls produced during the 5 min playback period, and the bottom panels **(C,D)** show calls produced during song bouts. Statistical significance for B71 **(B,D)** is depicted with letters based on pairwise comparison of emmeans (all *p*’s < 0.001). Statistical significance is not presented for Song W72 because only four females called leaving the model underfitted.

Female calling responses differed by Motif Condition (*# Calls* glmer χ^2^ (3) = 174.08, *p* < 0.001; *Call Rate* lmer Playback χ^2^ (3) = 8.44, *p* = 0.038); however, there was also an effect of male’s song (B71 > W72 glmer χ^2^ (1) = 40.37, *p* < 0.001) (Figure 4). Overall, females called more in response to B71 male’s song: only four out of 10 females called in response to W72’s song, whereas eight out of the 10 females called in response to B71’s song. When we analyzed the effect of motif condition on calling to B71 and W72 songs separately, motif condition appeared to be a significant factor in female responses (#calls) to both songs (Figures 4C,D: glmer *Song W72* χ^2^ (3) = 32.08, *p* < 0.001; *Song B71* χ^2^ (3) = 170.07, *p* < 0.001). However, very few females called in response to W72, meaning there was very little variation in that model.

Both the effects of Motif Condition and Male Song on female calling responses are evident regardless of whether we analyze the results using calls produced during the 5 min of playback (total # calls or call rate) (Figure 4), or focus on calls specifically produced within song bouts (glmer *Motif Condition* χ^2^ (3) = 113.91, *p* < 0.001). Again, female responses to B71 and W72 songs differed (*Male Song* χ^2^ (1) = 32.51, *p* < 0.001; effect of Motif Condition for *Song W72* χ^2^ (3) = 8.04, *p* = 0.045; *Song B71* χ^2^ (3) = 110.77, *p* < 0.001). Furthermore, the effect of motif condition was not evident for call rate pre or post playback (*Pre* χ^2^ (3) = 2.69, *p* = 0.442; *Post* χ^2^ (3) = 4.75, *p* = 0.191).

#### Females reduced calling in response to reversed syllables

Females responded very little or not at all to motifs where the syllables were reversed (Figure 4). For Song W72, there was no calling during the REVERSAL condition. For Song B71, there was a significant decrease in calling (emmeans FIXED – REVERSAL *p* < 0.001; RENDITION – REVERSAL *p* < 0.001; SHUFFLED – REVERSAL *p* < 0.001). This effect is present when comparing total calls during the 5 min playback and when focusing on calls produced within song bouts (emmeans FIXED – REVERSAL *p* < 0.001; RENDITION – REVERSAL *p* < 0.001; SHUFFLED – REVERSAL *p* < 0.001).

#### Females differentially call to naturally occurring acoustic variation across syllable renditions

Females also modulated their calling based on naturally occurring acoustic variation across renditions of syllables (Figure 4). For Song B71, females called significantly less for the RENDITION condition compared to the SHUFFLED and FIXED conditions (pairwise emmeans *p*’s < 0.001). Again, this effect is present when comparing total calls during the 5 min playback or when focusing on calls only during the songs (pairwise emmeans *p*’s < 0.001). Interestingly, there appeared to be an effect in the opposite direction for W72 (RENDITION – SHUFFLED *p* = 0.013). In contrast, females called similarly to SHUFFLED and FIXED playback conditions for B71 (*# Calls* FIXED – SHUFFLED *p* = 0.769; *# Calls within song bouts* FIXED – SHUFFLED *p* = 0.674).

### Females habituate to male song over repeated sessions (Experiment 3)

#### Female calling responses to motif conditions presented in a single playback (Exp 3a)

**Figure 5:**
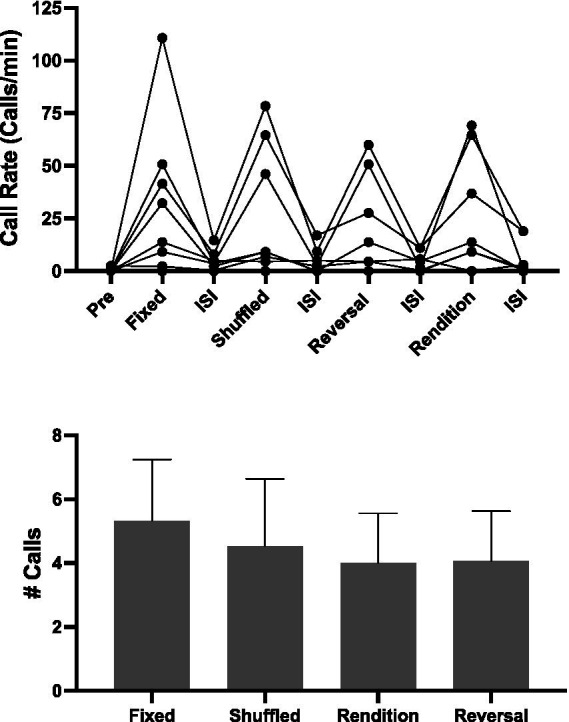
Female responses to motif conditions presented in a single playback. **(A)** Call rate during each silence and motif condition. Note the different durations: Pre (60 s), Song bouts (6.5 s), Interstimulus Silences (45 s), Post (30 s). Also note that the order of presentations was counterbalanced across individuals, so the order of the playback in the graph is not the order each bird received the stimuli. The order of Motif Conditions along with their subsequent interstimulus silence is presented in an order consistent with the other experiments. Most females increased their call rate in response to playback (***p < 0.001). **(B)** Despite that their calling in response to playback is elevated, there was no effect of motif condition on female responses.

In this experiment, we used Song B71 because it elicited more female responses in Exp 2. Here we attempted to replicate the results with a new set of females, but this time we included all the conditions in one playback. Our hope was that including all the conditions in one playback would decrease day-to-day variation in female responses. However, we were unsure whether comparing calling to motif conditions that change rapidly (~ every 1 min) in a playback session would yield selective responses. Overall, even on this short timescale, females did increase their call rate during song playbacks relative to inter-stimulus intervals (ISI) (Figure 5; lmer χ^2^ (3) = 52.18, *p* < 0.001).

Of the eight females, seven called in this experiment. However, there was no evidence for modulation of calling based on Motif Condition (*# Calls*, glmer χ^2^ (3) = 3.65, *p* = 0.302; *Call Rate,* lmer χ^2^ (3) = 2.76, *p* = 0.431). Furthermore, there was no difference between calling in response to reversals compared to any of the other playback conditions.

#### Females decrease calling to REVERSALS vs. FIXED conditions presented in a single playback (Exp 3b)

**Figure 6:**
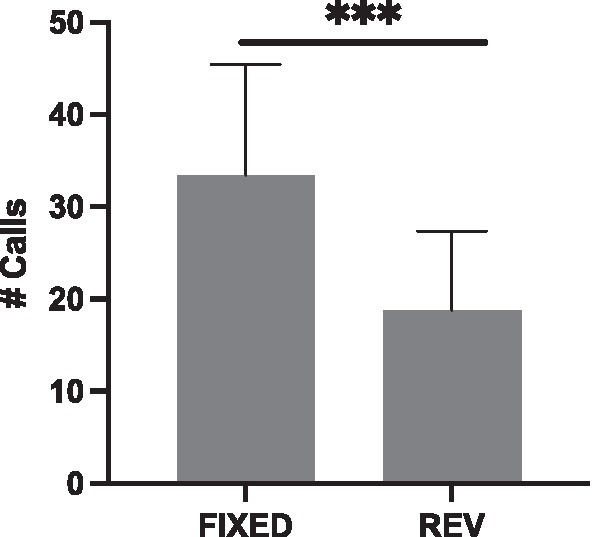
Females called less to REVERSALS (REV) when both motif conditions were placed in the same playback (***p < 0.001).

Again, using Song B71, we tried to replicate the effect of reversals on female calling responses by using two, instead of four, motif conditions per playback (FIXED and REVERSAL) and by switching motif conditions halfway through (Figure 6). With these adjustments, we did see that females called less in response to the REVERSAL relative to the FIXED condition (glmer χ^2^ (1) = 32.21, *p* < 0.001); however, this was driven by an effect in the first Session (*Session 1* glm χ^2^ (1) = 66.98, *p* < 0.001; *Session 2* glm χ^2^ (1) = 0.75, *p* = 0.386).

#### Visual component did not increase call response (Exp 3b)

While we included a visual component with the goal of increasing female responsivity, it appears to have decreased female calling (*Session 1 + Session 2* Mean ± SEM: *Playback only* = 35.25 ± 14.50, *Playback + Visual* = 16.86 ± 8.85. glmer χ^2^ (1) = 49.97, *p* < 0.001). This effect was driven by an effect in Session 1 (*Session 1: Playback only* = 51.75 ± 22.75, *Playback + Visual* = 12.25 ± 9.66. glm χ^2^ (1) = 104.90, *p* < 0.001; *Session 2 only* χ^2^ (1) = 0.75, *p* = 0.386).

#### Females habituate to male stimuli (Exps 3a and 3b)

**Figure 7:**
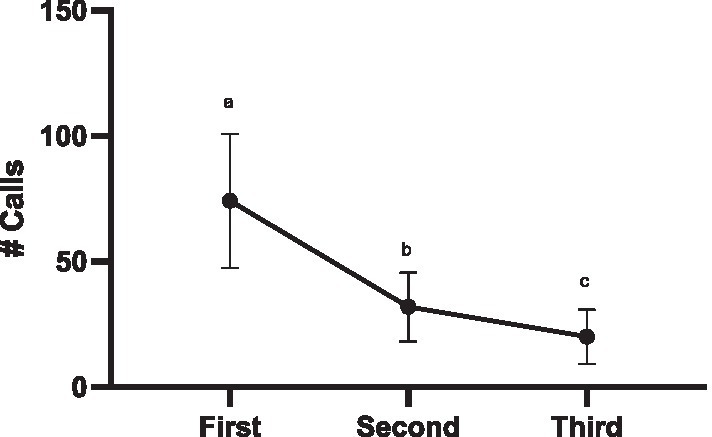
Decreased calling following repeated exposure: Exp 3 (first session) and Exp 3b Session 1 and Session 2 (second and third session). Statistical significance is depicted with letters based on pairwise comparison of emmeans (all *p*’s < 0.001).

In this experiment, the eight females were exposed to the same stimuli three times over 3 days. Over the course of those three sessions, females decreased their call responses (glmer χ^2^ (2) = 277.60, *p* < 0.001; all pairwise comparisons *p* < 0.001) (Figure 7). For the first two presentations, 7 out of 8 females responded (*First Session* min-max calls produced 21–200 calls, Mean ± SEM = 74.25 ± 25.63; *Second Session* 1–109 calls, 32.64 ± 13.66). By the third session only 4 females responded (*Third Session* 6–69 calls, 20.13 ± 10.78).

### Distorting only a few syllables within a song bout is behaviorally relevant (Experiment 4)

**Figure 8:**
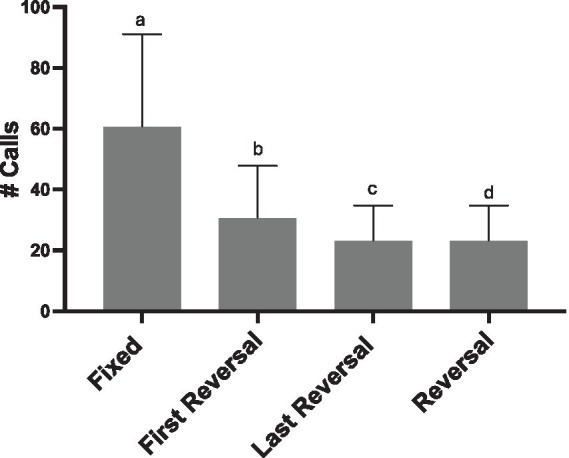
Effect of three reversal conditions on female calling (REVERSAL, where all syllables in a song are reversed; FIRST REVERSAL, only the syllables in the first motif are reversed, and LAST REVERSAL only the syllables in the last motif are reversed). Not only do females decrease calling in response to all syllables in a song bout being reversed, but they reduce calling if only 1/8th of the syllables at the beginning or the end are reversed (FIRST and LAST REVERSAL). Statistical significance is depicted with letters based on pairwise comparison of emmeans (all *p*’s < 0.020).

To determine how sensitive females are to syllable manipulations, we examined the effect of reversing only a portion of syllables within a song bout. We found that for all REVERSAL conditions females called less than they did in response to the FIXED condition (*Total # Calls*: glmer χ^2^ (3) = 276.64, *p* < 0.001; all pairwise comparisons *p* < 0.020; Figure 8). This main effect was seen for calls throughout the playback period, but also for calls produced within songs (*# Calls within Song:* glmer χ^2^ (3) = 44.25, *p* < 0.001).

#### Repeated exposure with the same male’s song decreased female calling

**Figure 9:**
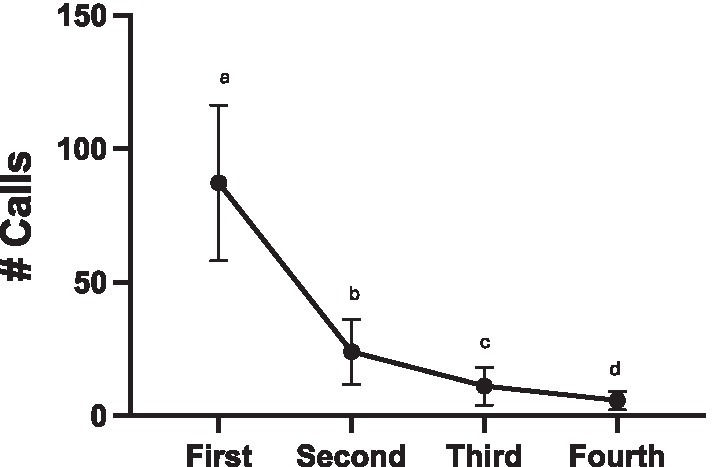
Decreased calling across repeated sessions within Exp 4. Every bird was tested on each of the four experimental conditions (FIXED, REVERSAL, FIRST REVERSAL, LAST REVERSAL) in separate playback sessions on separate days. The order of presentation was counter balanced. Emmeans pairwise comparisons (*p* < 0.001 *** except third-fourth *p* < 0.01**).

Even with songs from 5 different males included in each stimulus, females decreased calling following each of the four sessions where they were exposed to stimuli (glmer χ^2^ (3) = 738.39, *p* < 0.001; all pairwise comparisons *p* < 0.001, except third-fourth *p* = 0.0015; Figure 9). In the first two sessions, 6 out of 8 females called (First session: min-max calls produced, 25–216 calls; Second session: 3–94 calls). In the third session 4 out of the 8 females called (min-max calls produced, 1–47 calls). By the fourth session only 3 out of the 8 females called (min-max calls produced, 8–27 calls).

## General discussion

Leveraging natural courtship dynamics, we used female calls in response to song to further our understanding of how females assess syllable level details within a song. Female calling responses offer a simple, yet powerful ethologically appropriate behavioral indicator of female interest to interact with a given male (Exp 1). Our results reveal how sensitive females are to manipulations of song syllables and illustrate that disruptions in only a portion of syllables can cause decreases in calling responses (Exp 2–4). However, our experiments also demonstrate how quickly females can habituate to hearing individual males (Exp 3–4). Altogether our findings raise new questions about how females assess syllable level features relative to global features and suggest that birds’ remarkable ability to discriminate acoustic fine structure plays a role in communication. Below, we also further discuss the strengths and challenges of this behavioral approach.

### Challenges with playback approach

Many different behavioral paradigms have been used to study female song preferences. While some of these studies have been conducted in the field (Mountjoy and Lemon, 1996; Searcy, 1984), the majority are conducted in the lab. The different lab paradigms have various strengths and weaknesses. Many paradigms require birds to undergo training, such as key pecking (Anderson, 2009; Le Maguer et al., 2021; Riebel, 2003; Riebel and Slater, 1998), air puff/aversive stimuli (Tokarev et al., 2017), and perch hopping (Anderson et al., 2014; Braaten and Reynolds, 1999; Howell et al., 2019; Sewall et al., 2018; Spencer et al., 2005). Training can allow for greater control over female behavior, and greater clarity in female decision-making. However, operant assays are also disconnected from natural behavioral interactions, are less ethologically relevant, and individual variation in cognitive and/or personality traits may artificially select who successfully completes the task. Other approaches leverage natural behavioral responses to passive playback, such as proximity (Nolan and Hill, 2004), copulation solicitation displays (Dunning et al., 2014; Searcy, 1984), or calls (Amy et al., 2015; Barr et al., 2021; Chen et al., 2017). There is some evidence that these behavioral responses similarly reflect female preferences (e.g. copulation solicitation displays and calling) (Dunning et al., 2014). Additionally, there is some evidence that females make consistent decisions about song and male attractiveness across paradigms; for example, passive playbacks (assessing phototaxis), operant tests, and interactions with a live male (Holveck and Riebel, 2007). However, there have only been a few studies investigating how similar/consistent female preferences are across behaviors and paradigms.

Even paradigms leveraging behavioral responses to passive playback, vary with the extent to which they reflect ethologically relevant aspects of social interactions during courtship and pairing. In zebra finches, courtship interactions are dynamic and require mutual adjustments between the male and female (D’Amelio et al., 2017; Hyland Bruno et al., 2021; Prior et al., 2020b). Over time, zebra finch pairs continue to have interactive vocal exchanges across social contexts (Boucaud et al., 2016; D’Amelio et al., 2017; Elie et al., 2010; Prior, 2020; Prior et al., 2020a). Arguably, for zebra finches the most ethologically appropriate response may be calls.

Considering the interactive and co-created nature of zebra finch courtship, it may in fact be unsurprising that females habituated so quickly to passive playback. Whereas a female call response may be ethologically relevant, the passive nature of song playback would quickly disrupt this social interaction. The results of both Exps 3 and 4 demonstrate the striking extent to which females decrease call responses across repeated sessions. Importantly, at the start of each experiment, females responded similarly (even though the same females were used in Exps 3 and 4). In Exp 3, it is possible that females were habituating to the individual stimuli (motif condition per song) because the same motif conditions were present but in a different order across the three sessions (Table 1). In Exp 4, we used songs from 5 different males so in each playback session females were hearing only two repetitions of that male’s song, and each playback session had a different motif condition (Table 1). Our hope was that this would decrease the habituation effect, and it is quite striking that it had no effect. Given that females can easily identify, discriminate and, remember more than 40 individuals (Elie and Theunissen, 2018; Geberzahn and Derégnaucourt, 2020; Geberzahn et al., 2021; Yu et al., 2020), we suspect that females are habituating to the identity of the males in the playback. However, it is also possible that females habituate to the nature of the paradigm. If this is the case, it is noteworthy that we saw recovery of robust behavior across the 2 weeks between Exps 3 and 4. Future research could directly test the extent to which females habituate to the paradigm vs. a given male.

### Behavioral relevance of syllable level details

Previous work has largely overlooked the potential significance of syllable level details within and across song bouts. Across our four experiments we provide multiple lines of evidence highlighting the importance of syllable level details. First, females called similarly to FIXED and SHUFFLED songs (Exp 2). Second, females responded very little to REVERSALS (Exp 2, 3b, and 4). Furthermore, females called very little to songs where only a small portion (1/8th) of the syllables were reversed (Exp 4). While these results are consistent with our perceptual experiments (Fishbein et al., 2021; Lawson et al., 2018; Prior et al., 2018; Stennette et al., 2023), this is an important step in demonstrating the behavioral significance of these syllable level details.

#### Female zebra finches are insensitive to syllable order

Given that male and female zebra finches are largely unable to discriminate manipulations of syllable order (shuffles) for unfamiliar songs (Fishbein et al., 2020; Lawson et al., 2018; Stennette et al., 2023), we predicted that females would respond similarly to these motif conditions. Consistent with our prediction, in Exp 2 we saw that females called similarly to SHUFFLED and FIXED songs. Based on our psychoacoustic results, one explanation for this effect is that females could not hear the differences between FIXED and SHUFFLED conditions because these were unfamiliar male’s songs and the order of syllables is not salient to zebra finches. Interestingly, zebra finches can become more sensitive to syllable order as they gain familiarity with stimuli either socially or via training (Lawson et al., 2018; Ning et al., 2023; Ten Cate and Spierings, 2019). Thus, an interesting follow up would be to test how females respond to syllable vs. sequence manipulations in their mate’s song, when presumably the relative salience of syllable order is higher than it is in our current experiment.

#### Song-specific responses to the rendition

Our recent research demonstrated that zebra finches are able to easily discriminate between natural syllable and motif renditions (Fishbein et al., 2021). B71 and W72 were songs used in that experiment; thus, while there may be variation in the extent to which renditions are discriminable, we can be confident our stimuli were easily discriminable. While it remains unclear what acoustic features zebra finches used to discriminate these near-identical renditions (Fishbein et al., 2021), in this study we show that females respond to these differences. This suggests that behaviorally relevant information is encoded in the acoustic fine structure of these vocalizations.

Interestingly, we found that for one male’s song, call rate was higher with the RENDITION condition, whereas for the other male, call rate was higher during the FIXED condition. If females prefer complexity in syllables, we might expect that females would consistently call less during the fixed motif playback than the varied rendition motif playback. Indeed, this has been suggested previously (Collins, 1999). Our results are not consistent with this notion. Instead, our results suggest that females are assessing males based on their ability to produce certain unknown features in song and/or based off high-quality rendition. Thus, we may expect that females are calling more during whichever playback condition had the highest quality renditions of a syllable, and that we are currently unaware of what makes a motif high quality. This highlights the importance of not assuming which songs are high quality, but rather asking females, as a recent study has done (Alam et al., 2024). Furthermore, additional experimentation is needed to determine the relative importance of syllable variation during a courtship context versus other social contexts (e.g., during pair maintenance).

#### Females call very little to reversals

The most robust effect of our motif conditions across our experiments was that female zebra finches call very little (or not at all) to syllable-reversed motifs. This is not surprising given that reversals are easily discriminable (Lawson et al., 2018), and are a significant manipulation which could disrupt the acoustic signatures that are behaviorally relevant. We included reversals because they are a profound syllable disruption (non-ethologically relevant), but still contain similar acoustic features, and allow us to maintain other species typical aspects of song.

Given the robust effect of reversals in Exp 2, we were able to use reversals in Exp 3b to test how the adjustments to our paradigm worked. In Exp 3, we included all four motif conditions in a single playback with only brief inter-song silences (~1 min). When we failed to replicate the results of Exp 2, especially for reversals, we suspected it was the design of the playback, that we too rapidly changed the motif conditions and/or included too many conditions in a short period of time. Therefore, we used only FIXED and REVERSAL conditions in Exp 3b, where the first half of the playback (~4 min) contained songs from one condition. Using this modification, we did replicate the decreased calling with reversals in Exp 2.

While it will surely be unsurprising to many that females called very little in response to REVERSALS, it is striking that females decrease calling similarly if every syllable in a song is reversed or if only a small portion of syllables are reversed. In Exp 4, we showed that females decreased their calling when every syllable is reversed, when only syllables in the first motif were reversed, and when only the syllables in the last motif were reversed. However, since every syllable in a motif was manipulated, it is unclear if this effect is driven by performance on a few key syllables or if females are attending to each syllable. An alternative hypothesis is that females were remembering across sessions that these males had disruptions in their song, and that the decreased responsivity in song was accumulated across song bouts. Future experiments could test the extent to which females remember poor experiences with a song vs. are sensitive to individual syllable manipulations.

## Conclusion

Leveraging natural courtship dynamics offers a powerful opportunity to gain insights into how females assess bird song. A huge advantage of using female calling is it reliably provides rapid responses using brief behavioral assays. In fact, our results suggest increased exposure to stimuli over a longer period or multiple sessions decreases the efficacy of the test. However, there are also challenges. In general, calling responses were low, highlighting the importance of considering methodological approaches to minimize the stress of the assay (e.g., minimizing handling, including additional habituation periods). Our results also highlight that the choice of stimuli shapes female responsivity. Unfortunately, reusing the same male’s song, to control for male-to-male differences in motifs, may need to be done sparingly. In the future, some best practices could be using songs from many males in stimuli, representing songs sparingly across sessions, leaving long periods of rest between sessions, and/or building the ‘habituation’ effect into experimental designs. Further studies could also investigate other solutions, such as using multi-modal stimuli (Varkevisser et al., 2022).

Bouts of birdsong often sound the same to the casual human listener. Altogether, our results align with growing evidence that syllable level details contain critical information for zebra finches (Geberzahn and Derégnaucourt, 2020; Geberzahn et al., 2021; Lawson et al., 2018; Prior et al., 2018; Stennette et al., 2023). Furthermore, this work provides evidence that females could be assessing mates based on syllable level details (Alam et al., 2024; Fishbein et al., 2021). Finally, our results are consistent with the hypothesis that birds’ exquisite sensitivity to the acoustic fine structure within a complex syllable may serve an important function in communication (Dooling and Prior, 2017).

## Data Availability

The raw data supporting the conclusions of this article will be made available by the authors, without undue reservation.

## References

[ref1] Alam D. Zia F. Roberts T. F. (2024). The hidden fitness of the male zebra finch courtship song. Nature 628, 117–121. doi: 10.1038/s41586-024-07207-4, PMID: 38509376 PMC11410162

[ref2] Amy M. Salvin P. Naguib M. Leboucher G. (2015). Female signalling to male song in the domestic canary, *Serinus canaria*. Royal Soc. Open Sci. 2:140196. doi: 10.1098/rsos.140196, PMID: 26064577 PMC4448791

[ref3] Anderson R. C. (2009). Operant conditioning and copulation solicitation display assays reveal a stable preference for local song by female swamp sparrows *Melospiza georgiana*. Behav. Ecol. Sociobiol. 64, 215–223. doi: 10.1007/s00265-009-0838-y

[ref4] Anderson R. C. Peters S. Nowicki S. (2014). Effects of early auditory experience on the development of local song preference in female swamp sparrows. Behav. Ecol. Sociobiol. 68, 437–447. doi: 10.1007/s00265-013-1658-7

[ref5] Barr H. J. Wall E. M. Woolley S. C. (2021). Dopamine in the songbird auditory cortex shapes auditory preference. Curr. Biol. 31, 4547–4559.e5. e4545. doi: 10.1016/j.cub.2021.08.005, PMID: 34450091

[ref6] Bates D. Machler M. Bolker B. Walker S. (2015). Fitting linear mixed-effects models using {lme4}. J. Stat. Softw. 67, 1–48. doi: 10.18637/jss.v067.i01

[ref7] Bateson P. P. G. (1983). Mate choice. New York: Cambridge University Press.

[ref8] Boucaud I. Mariette M. Villain A. Vignal C. (2016). Vocal negotiation over parental care? Partners adjust their time spent incubating based on their acoustic communication at the nest. Biol. J. Linnean Soc. 117, 322–336. doi: 10.1111/bij.12705

[ref9] Braaten R. F. Reynolds K. (1999). Auditory preference for conspecific song in isolation-reared zebra finches. Anim. Behav. 58, 105–111. doi: 10.1006/anbe.1999.1134, PMID: 10413546

[ref10] Byers B. E. Kroodsma D. E. (2009). Female mate choice and songbird song repertoires. Anim. Behav. 77, 13–22. doi: 10.1016/j.anbehav.2008.10.003

[ref11] Catchpole C. Slater P. J. B. (2008). Bird song: Biological themes and variations. Cambridge: Cambridge Univ Press.

[ref12] Chen Y. Clark O. Woolley S. C. (2017). Courtship song preferences in female zebra finches are shaped by developmental auditory experience. Proc. R. Soc. B Biol. Sci. 284:20170054. doi: 10.1098/rspb.2017.0054, PMID: 28539523 PMC5454257

[ref13] Clayton N. S. (1988). Song discrimination learning in zebra finches. Anim. Behav. 36, 1016–1024. doi: 10.1016/S0003-3472(88)80061-7

[ref14] Clayton N. S. Pröve E. (1989). Song discrimination in female zebra finches and Bengalese finches. Anim. Behav. 38, 352–354. doi: 10.1016/S0003-3472(89)80096-X

[ref15] Coleman M. Fortune E. (2018). Duet singing in plain-tailed wrens. Curr. Biol. 28, R643–R645. doi: 10.1016/j.cub.2018.02.066, PMID: 29870698

[ref16] Collins S. A. (1999). Is female preference for male repertoires due to sensory bias? Proc. R. Soc. Lond. Ser. B Biol. Sci. 266, 2309–2314.

[ref17] D’Amelio P. B. Trost L. ter Maat A. (2017). Vocal exchanges during pair formation and maintenance in the zebra finch (*Taeniopygia guttata*). Front. Zool. 14:13. doi: 10.1186/s12983-017-0197-x, PMID: 28250800 PMC5324246

[ref18] Dooling R. J. Prior N. H. (2017). Do we hear what birds hear in birdsong? Anim. Behav. 124, 283–289. doi: 10.1016/j.anbehav.2016.10.012, PMID: 29628517 PMC5884127

[ref19] Dunning J. L. Pant S. Bass A. Coburn Z. Prather J. F. (2014). Mate choice in adult female Bengalese finches: females express consistent preferences for individual males and prefer female-directed song performances. PLoS One 9:e89438. doi: 10.1371/journal.pone.0089438, PMID: 24558501 PMC3928452

[ref20] Elie J. E. Mariette M. M. Soula H. A. Griffith S. C. Mathevon N. Vignal C. (2010). Vocal communication at the nest between mates in wild zebra finches: a private vocal duet? Anim. Behav. 80, 597–605. doi: 10.1016/j.anbehav.2010.06.003

[ref21] Elie J. E. Theunissen F. E. (2018). Zebra finches identify individuals using vocal signatures unique to each call type. Nat. Commun. 9:4026. doi: 10.1038/s41467-018-06394-9, PMID: 30279497 PMC6168511

[ref22] Fishbein A. R. Idsardi W. J. Ball G. F. Dooling R. J. (2020). Sound sequences in birdsong: how much do birds really care? Philos. Trans. R. Soc. B 375:20190044. doi: 10.1098/rstb.2019.0044, PMID: 31735149 PMC6895548

[ref23] Fishbein A. R. Prior N. H. Brown J. A. Ball G. F. Dooling R. J. (2021). Discrimination of natural acoustic variation in vocal signals. Sci. Rep. 11:916. doi: 10.1038/s41598-020-79641-z, PMID: 33441711 PMC7807010

[ref24] Forstmeier W. Birkhead T. R. (2004). Repeatability of mate choice in the zebra finch: consistency within and between females. Anim. Behav. 68, 1017–1028. doi: 10.1016/j.anbehav.2004.02.007

[ref25] Geberzahn N. Derégnaucourt S. (2020). Individual vocal recognition in zebra finches relies on song syllable structure rather than song syllable order. J. Exp. Biol. 223:jeb 220087. doi: 10.1242/jeb.220087, PMID: 32253282

[ref26] Geberzahn N. Zsebők S. Derégnaucourt S. (2021). Auditory perception of self and others in zebra finches: evidence from an operant discrimination task. J. Exp. Biol. 224:jeb 233817. doi: 10.1242/jeb.233817, PMID: 33653723

[ref27] Hauber M. E. Campbell D. L. M. Woolley S. M. N. (2010). The functional role and female perception of male song in Zebra finches. Emu-Austral Ornithol. 110, 209–218. doi: 10.1071/MU10003

[ref28] Holveck M.-J. Riebel K. (2007). Preferred songs predict preferred males: consistency and repeatability of zebra finch females across three test contexts. Anim. Behav. 74, 297–309. doi: 10.1016/j.anbehav.2006.08.016

[ref29] Howell C. Anderson R. Derryberry E. (2019). Female cognitive performance and mass are correlated with different aspects of mate choice in the zebra finch (*Taeniopygia guttata*). Anim. Cogn. 22, 1085–1094. doi: 10.1007/s10071-019-01299-6, PMID: 31401761

[ref30] Hyland Bruno J. Jarvis E. D. Liberman M. Tchernichovski O. (2021). Birdsong learning and culture: analogies with human spoken language. Ann. Rev. Linguist. 7, 449–472. doi: 10.1146/annurev-linguistics-090420-121034

[ref31] Lawson S. L. Fishbein A. R. Prior N. H. Ball G. F. Dooling R. J. (2018). Relative salience of syllable structure and syllable order in zebra finch song. Anim. Cogn. 21, 467–480. doi: 10.1007/s10071-018-1182-2, PMID: 29766379 PMC6438364

[ref32] Le Maguer L. Derégnaucourt S. Geberzahn N. (2021). Female preference for artificial song dialects in the zebra finch (*Taeniopygia guttata*). Ethology 127, 537–549. doi: 10.1111/eth.13159

[ref33] Lenth R. V. (2024). emmeans: estimated marginal means, aka least-squares means. R package version 1.10.6-090003. Available at: https://github.com/rvlenth/emmeans

[ref34] Mountjoy D. J. Lemon R. E. (1996). Female choice for complex song in the European starling: a field experiment. Behav. Ecol. Sociobiol. 38, 65–71. doi: 10.1007/s002650050218

[ref35] Nagle L. Kreutzer M. Vallet E. (2002). Adult female canaries respond to male song by calling. Ethology 108, 463–472. doi: 10.1046/j.1439-0310.2002.00790.x

[ref36] Ning Z.-Y. Honing H. Ten Cate C. (2023). Zebra finches (*Taeniopygia guttata*) demonstrate cognitive flexibility in using phonology and sequence of syllables in auditory discrimination. Anim. Cogn. 26, 1161–1175. doi: 10.1007/s10071-023-01763-4, PMID: 36934374 PMC10345033

[ref37] Nolan P. M. Hill G. E. (2004). Female choice for song characteristics in the house finch. Anim. Behav. 67, 403–410. doi: 10.1016/j.anbehav.2003.03.018

[ref38] Perez E. C. Fernandez M. S. A. Griffith S. C. Vignal C. Soula H. A. (2015). Impact of visual contact on vocal interaction dynamics of pair-bonded birds. Anim. Behav. 107, 125–137. doi: 10.1016/j.anbehav.2015.05.019

[ref39] Prior N. H. (2020). What’s in a moment: what can be learned about pair bonding from studying moment-to-moment behavioral synchrony between partners? Front. Psychol. 11:1370. doi: 10.3389/fpsyg.2020.01370, PMID: 32848962 PMC7417665

[ref40] Prior N. H. Fernandez M. S. A. Soula H. A. Vignal C. (2019). Water restriction influences intra-pair vocal behavior and the acoustic structure of vocalisations in the opportunistically breeding zebra finch (*Taeniopygia guttata*). Behav. Process. 162, 147–156. doi: 10.1016/j.beproc.2019.02.007, PMID: 30825505

[ref41] Prior N. H. Smith E. Dooling R. J. Ball G. F. (2020a). Familiarity enhances moment-to-moment behavioral coordination in zebra finch (*Taeniopygia guttata*) dyads. J. Comp. Psychol. 134, 135–148. doi: 10.1037/com0000201, PMID: 31647250 PMC7180088

[ref42] Prior N. H. Smith E. Dooling R. J. Ball G. F. (2020b). Monogamy in a moment: how do brief social interactions change over time in pair-bonded zebra finches (*Taeniopygia guttata*)? Integr. Organ. Biol. 2:obaa034. doi: 10.1093/iob/obaa034, PMID: 33791572 PMC7810576

[ref43] Prior N. H. Smith E. Lawson S. Ball G. F. Dooling R. J. (2018). Acoustic fine structure may encode biologically relevant information for zebra finches. Sci. Rep. 8:6212. doi: 10.1038/s41598-018-24307-0, PMID: 29670131 PMC5906677

[ref44] Prior N. H. Soma K. K. (2015). Neuroendocrine regulation of long-term pair maintenance in the monogamous zebra finch. Horm. Behav. 76, 11–22. doi: 10.1016/j.yhbeh.2015.04.014, PMID: 25935729

[ref45] Riebel K. (2000). Early exposure leads to repeatable preferences for male song in female zebra finches. Proceedings of the Royal Society of London. Ser. B Biol. Sci. 267, 2553–2558. 10.1098/rspb.2000.1320 PMC1690843 11197134

[ref46] Riebel K. (2003). Developmental influences on auditory perception in female zebra finches-is there a sensitive phase for song preference learning? Anim. Biol. 53, 73–87. doi: 10.1163/157075603769700304

[ref47] Riebel K. Slater P. J. (1998). Testing female chaffinch song preferences by operant conditioning. Anim. Behav. 56, 1443–1453. doi: 10.1006/anbe.1998.0933, PMID: 9933541

[ref48] Rose E. M. Prior N. H. Ball G. F. (2022). The singing question: re-conceptualizing birdsong. Biol. Rev. 97, 326–342. doi: 10.1111/brv.12800, PMID: 34609054

[ref49] Rosenthal G. G. (2017). Mate choice: the evolution of sexual decision making from microbes to humans: Princeton New Jersey: Princeton University Press.

[ref50] Searcy W. A. (1984). Song repertoire size and female preferences in song sparrows. Behav. Ecol. Sociobiol. 14, 281–286. doi: 10.1007/BF00299499

[ref51] Sewall K. B. Anderson R. C. Soha J. A. Peters S. Nowicki S. (2018). Early life conditions that impact song learning in male zebra finches also impact neural and behavioral responses to song in females. Dev. Neurobiol. 78, 785–798. doi: 10.1002/dneu.22600, PMID: 29675841 PMC6195868

[ref52] Spencer K. Wimpenny J. Buchanan K. Lovell P. G. Goldsmith A. Catchpole C. (2005). Developmental stress affects the attractiveness of male song and female choice in the zebra finch (*Taeniopygia guttata*). Behav. Ecol. Sociobiol. 58, 423–428. doi: 10.1007/s00265-005-0927-5

[ref53] Stennette K. A. Fishbein A. Prior N. Ball G. F. Dooling R. J. (2023). Sound order discrimination in two species of birds–Taeniopygia guttata and *Melopsittacus undulatus*. J. Comp. Psychol. 137, 29–37. doi: 10.1037/com0000340, PMID: 36931835

[ref54] Strong J. (2017). ML83468711 Chestnut-backed Antbird Poliocrania exsul. Cornell Lab of Ornithology Macaulay Library. Available at: https://macaulaylibrary.org/asset/83468711

[ref55] Sueur J. Aubin T. Simonis C. (2008). Seewave, a free modular tool for sound analysis and synthesis. Bioacoustics 18, 213–226. doi: 10.1080/09524622.2008.9753600

[ref56] Ten Cate C. Spierings M. (2019). Rules, rhythm and grouping: auditory pattern perception by birds. Anim. Behav. 151, 249–257. doi: 10.1016/j.anbehav.2018.11.010

[ref57] Tokarev K. Bruno J. H. Ljubičić I. Kothari P. J. Helekar S. A. Tchernichovski O.. (2017). Sexual dimorphism in striatal dopaminergic responses promotes monogamy in social songbirds. Elife 6:e25819. doi: 10.7554/eLife.25819, PMID: 28826502 PMC5584986

[ref58] Varkevisser J. Simon R. Mendoza E. Scharff C. Halfwerk W. Riebel K. (2022). Multimodal cues in songbird vocal learning provide perspective on discrepancies between live and audio-only tutoring. Seeing voices: the role of multimodal cues in vocal learning, vol. 21.

[ref59] Wall E. M. Woolley S. C. (2024). Social experiences shape song preference learning independently of developmental exposure to song. Proceedings B 291:20240358. doi: 10.1098/rspb.2024.0358, PMID: 38835281 PMC11285830

[ref60] Woodgate J. L. Mariette M. M. Bennett A. T. D. Griffith S. C. Buchanan K. L. (2012). Male song structure predicts reproductive success in a wild zebra finch population. Anim. Behav. 83, 773–781. doi: 10.1016/j.anbehav.2011.12.027

[ref61] Woolley S. C. Doupe A. J. (2008). Social context–induced song variation affects female behavior and gene expression. PLoS Biol. 6:e62. doi: 10.1371/journal.pbio.0060062, PMID: 18351801 PMC2267820

[ref62] Woolley S. C. Woolley S. M. (2020). Integrating form and function in the songbird auditory forebrain. Neuroethol. Birdsong, 71, 127–155. doi: 10.1007/978-3-030-34683-6_5

[ref63] Yu K. Wood W. Theunissen F. (2020). High-capacity auditory memory for vocal communication in a social songbird. Sci. Adv. 6:eabe0440. doi: 10.1126/sciadv.abe0440, PMID: 33188032 PMC7673746

[ref64] Zann R. A. (1996). The zebra finch: a synthesis of field and laboratory studies. New York: Oxford University Press.

